# A Study of prevalence of *Shigella* species and antimicrobial resistance patterns in paediatric medical center, Ahvaz, Iran

**Published:** 2017-10

**Authors:** Roya Nikfar, Ahmad Shamsizadeh, Marjan Darbor, Soheila Khaghani, Mina Moghaddam

**Affiliations:** 1 Infectious and Tropical Diseases Research Center, Health Research Institute, Ahvaz Jundishapur University of Medical Sciences, Ahvaz, Iran; 2 Department of Pediatrics, Aboozar Children’s Hospital, Ahvaz Jundishapur University of Medical Sciences, Ahvaz, Iran

**Keywords:** Anti-microbial resistance, *Shigella*, Children

## Abstract

**Background and Objectives:**

*Shigella* infections are one of the major causes of diarrhea worldwide, and especially in developing countries. Antimicrobial resistance has complicated the empirical treatment. The aim of this study was to define the clinical and antibiotic resistance patterns of *Shigella* gastroenteritis cases.

**Materials and Methods:**

Stool samples of patients with diarrhea and fever diagnosed with shigellosis were collected, from June 2013 to May 2014 at Abuzar Hospital, Iran. All samples were cultured for *Shigella* spp on selective and differential media. *Shigella* isolates were evaluated for antimicrobial resistance.

**Results:**

Among 193 *Shigella* isolates, *S. flexneri* (64.8%) was the predominant species followed by *S. sonnei* (32.6%). The most frequent antibiotic resistance observed, was towards co-trimoxazole (89%), ampicillin (77%) and ceftriaxone (51%) and the lowest resistance were seen in ciprofloxacin (1.5%), azithromycin (7%).

**Conclusion:**

Due to the high resistance to ceftriaxone, this drug is not recommended as an empirical therapy for shigellosis. However, azithromycin should be used as the first-line treatment for paediatric patients, suffering from shigellosis and ciprofloxacin can be used as an alternative.

## INTRODUCTION

Diarrheal is a leading cause of death and disability in infants and children, worldwide. It has been estimated that 2–4 billion episodes of infectious diarrhea occur annually in developing countries, leading to 3–5 million deaths that is the highest number of deaths in children less than 5 years. Among the bacteria, *Shigella* has long been recognized as an important human pathogen, especially in developing countries with poor sanitation and no hygienic water resources (1). Resistance of *Shigella* to antibiotics is a growing problem in the countries of southeast Asia, Africa and south America (2). Although infection with *Shigella* can occur at any age, but it is more common in the second and third years of life (3). Like other family members of *Enterobacteriaceae*, *Shigella* is classified in four serogroups, based on the biochemical and serological characteristics: *Shigella dysenteriae, Shigella flexneri, Shigella boydii* and *Shigella sonnei*. Appropriate antibiotic treatment reduces the duration of symptoms and prevents life-threatening complications. Moreover, it shortens the time of excretion of pathogens in the stool and reduces the spread of infection. Unfortunately during recent decades, uncontrolled use of drugs has led to the creation of resistance to common and low cost antibiotics (3). Multidrug–resistant (MDR) shigellosis has been reported in different parts of the world (4, 5).

Considering the importance of antibiotics, in the treatment of shigellosis and differences in the epidemiology of anti-microbial susceptibility patterns of *Shigella* and also the high rates of its occurrence in Khuzestan province, this study has evaluated the clinical properties of antibiotic resistance to *Shigella* species in this region.

## MATERIALS AND METHODS

### Study area and period.

From June 2013 to May 2014, stool samples from patients with colitis were collected in Abuzar Hospital in Ahvaz, in the southwest of Iran. Ahvaz is the seat of Khuzestan province, where the weather is extremely hot during the year. Abuzar Hospital is the main Children’s Medical Center in this region. Children under the age of 12 were enrolled in this study. The required information was extracted from the patient’s records. Demographic data were collected, by filling out questionnaires. Information on (fever, bloody diarrhea, vomiting, neurological manifestations, seizure, loss of consciousness, drowsiness, coma, and the rate of dehydration was collected for all patients.

### Culture and biochemical tests.

A fresh fecal specimen was collected from patients and immediately transported to the laboratory within one hour of collection. All specimens were cultured on xylose lysine deoxycholate agar (XLD) (Merck, Germany) and MacConkey Agar (MAC) (Merck, Germany), using a swab and subsequently incubated at 37°C for 24 hours. Specimens were placed in Selenite F enrichment broth (Oxoid), incubated at 37°C for 24 hours. Subsequently subcultured onto Xylose-Lysine Deoxycholate agar (XLD) agar and incubated at 37°C for 18–24 hours. Red colonies suspicious to *Shigella*, on XLD agar were further identified by biochemical tests using suitable media such as Triple sugar Iron Agar (TSI) for carbohydrate fermentation test, Urea agar for urea utilization test, tryptophan broth for Indole test, Simmon Citrate agar for citrate utilization, SIM media for motility test and Lysine agar for lysine utilization test (6). *Shigella* isolates were sero-grouped by the slide agglutination test, using commercially available antisera (Baharafshan Co. Iran).

### Antimicrobial susceptibility testing.

Disk diffusion method was applied to determine the antibiotic susceptibility pattern of the *Shigella* isolates on Muller-Hinton agar (Merck, Germany) plates, according to the guidelines of Clinical Laboratory Standards Institute (CLSI) (7). The antimicrobial discs used in the study were ampicillin (10 μg), azithromycin (15μg), ceftriaxone (30μg), ciprofloxacin (5μg), nalidixic acid (30μg) co-trimoxazole (1.25/23.75ug). All antibiotics were obtained from Mast Ltd., UK. A standard inoculum equaled to 0.5 McFarland, was swabbed on Muller-Hinton agar. Antibiotic discs were distributed, after drying the plate for 3–5 minutes and incubation at 37°C for 24 hours. *E. coli* ATCC 25922 was used as control strain for the susceptibility tests. Data were recorded and analyzed using SPSS version 20 for windows. Chi-square test was used for comparisons. P-value of <0.05 was considered statistically significant.

## RESULTS

The results of this study detected 193 *Shigella* isolates in children hospitalized with gastroenteritis between 2013 to 2014, in Abuzar Hospital. *Shigella* species isolated based on patients’ stool culture were: *S. flexneri* in 125 patients (64.8%), *S. sonnei* in 63 patients (32.6%), *S. boydii* in 4 patients (2.1%) and *S. dysenteriae* in one patient (0.5%). *S. flexeneri* had the highest and *S. dysenteriae* had the lowest prevalence, among children with shigellosis (Table 1).

**Table 1.** Distribution of *Shigella* species in children during 2012 and 2013, in Ahvaz

***Shigella* Group**	**N (%)**
*S. flexneri*	125 (64.8%)
*S. sonnei*	63 (32.6%)
*S. boydii*	4 (2.1%)
*S. dysenteriae*	1 (0.5%)
Total	193

Of the 193 patients, 107 (55.4%) and 86 (44.6%) were males and females, respectively. There was no significant association between gender and *Shigella* infection (p > 0.05). *S. flexneri* isolates were mostly identified in males, whereas *S. boydii* was not isolated in either male or female. None of the females were infected with *S. dysenteriae* (Table 2). *Shigella* was frequently observed in patients 2–4 years of age. In general, the mean age of patients was two years and 9 months (Table 3). This study showed that in terms of clinical symptoms, 96 percent of patients had fever, and 32% had neurological symptoms (Table 4). The highest frequent resistance was 89% and 77% to co-trimoxazole and ampicillin, respectively. Highest resistance to co-trimoxazole was seen in samples infected with *S. flexneri*, while highest resistance to nalidixic acid was seen in specimens infected with *S. sonnei* (Table 5). In this study, 116 cases (57.5%) of *Shigella* isolates, which were simultaneously resistant to ampicillin and co-trimoxazole. In addition 81 (41.9%) of cases were resistant to ampicillin, ceftriaxone, co-trimoxazole and azithromycin. Although five cases (2.5%) were simultaneously resistant to ampicillin, ciprofloxacin, co-trimoxazole and azithromycin.

**Table 2.** Distribution of *Shigella* serogroups in children based on gender during 2012 and 2013, in Ahvaz

**Gender**	***S. flexneri* N (%)**	***S. sonnei* N (%)**	***S. boydii* N (%)**	***S. dysenteriae* N (%)**	**Total N (%)**	**P-value**
Male	68 (63.6%)	38 (35.5%)	0	1 (0.9%)	107 (55.4%)	0.09
Female	57 (66.3%)	25 (29.1%)	4 (4.7%)	0	86 (44.6%)	

**Table 3.** Distribution of *Shigella* in children, according to the age group, during 2012 and 2013 in Ahvaz

**Age**	**(%)**
< 2 years	57 (29.53)
2–4 years	72 (37.31)
≥4 years	64 (33.16)
Total	193 Cases

**Table 4.** Comparison of clinical symptoms of shigellosis in children during 2012 and 2013, in Ahvaz

**Clinical Symptom**	***S. flexneri***	***S. sonnei***	**P-value**
Bloody diarrhea	67 (53.6%)	31 (47.6%)	0.4
fever	119 (95.2%)	63 (100%)	0.7
vomiting	100 (80%)	49 (77.7%)	0.8
paroxysm	13 (10.4%)	7 (11.1%)	0.4
decreased level of consciousness	5 (4%)	2 (3%)	0.7
Coma	0 (0%)	0 (0%)	0
Total	125 cases	63 cases	

**Table 5.** Proportion of *Shigella* serogroups isolates resistant to antibiotics in children during 2012 and 2013, in Ahvaz

**Antibiotics**	***S. flexneri* N (%)**	***S. sonnei* N (%)**	***S. boydii* N (%)**	***S. dysenteriae* N (%)**	**Total N (%)**
**Co-trimoxazole**	109 (87.2%)	60 (95.2%)	3 (75%)	0	172 (89%)
**Ampicillin**	112 (89%)	36 (57.1%)	0	1 (100%)	149 (77%)
**Ceftriaxone**	74 (59.2%)	24 (38.1%)	1 (25%)	0	99 (51%)
**Nalidixic acid**	9 (7.2%)	14 (22.2%)	2 (50%)	0	25 (12%)
**Azithromycin**	7 (5.6%)	8 (12.7%)	0	0	15 (7%)
**Gentamicin**	2 (1.6%)	1 (1.59%)	0	0	3 (1.5%)
**Ciprofloxacin**	1 (0.8%)	2 (3.2%)	0	0	3 (1.5%)

## DISCUSSION

Diarrheal diseases are a public health problem among children worldwide, especially in developing countries and are considered as one of the causes of death, among them. The *Shigella* species has been considered as a potential factor in infectious diarrheas (1).

The current study investigated 193 cases of shigellosis in children with gastroenteritis, in Abuzar Hospital. Among the isolated species, *S. flexneri* was predominant. A three-year study (2008–2010) in this hospital had previously achieved the same results (8). Another study from Iran, reported *S. flexneri* as the common isolated species of *Shigella* (9). *S. flexneri* have been reported as the most common strain in some countries such as: Vietnam, Pakistan and Peru. (4, 5, 10). Some papers from Iran have reported *S. soneii* as the dominant serotype (11, 12). Although, Ahvaz is an industrial city, but most patients referring to the indicated hospital, are from rural areas. It is not clear whether the level of health has diminished or distribution of *Shigella* serotypes is very heterogeneous. Among the 193 children with shigellosis, 107 patients were males and 86 patients were females. This was consistent with the results of Zamani et al. (13). There is no correlation between gender and Shigellosis. Almost all patients in this study, aged from two to four. Most patients in a simillar study were 2 to 5 years old (13). Our study showed that in terms of clinical symptoms 96% of patients had fever, 79% had nausea and vomiting, 53% had bloody diarrhea and 32% had neurological symptoms. Coma was not observed in any of the patients. Sangeetha et al. described the prevalence of the symptoms as bloody diarrhea and mucus (58%), fever (51%), vomiting (18%) and seizures (9%) (14). Except for fever and vomiting, clinical symptoms were consistent with the results of our study. Perhaps the reason for this difference is overexpression of symptoms by parents when clinical signs were being recorded. In a previous study, in Abuzar Hospital bloody diarrhea was reported less than our study (9.23%), although neurological symptoms were reported more frequently than our results (9.39%) (15). Seizures was mostly reported in some studies (13). In this study, no significant difference in clinical symptoms was observed between patients with *S. flexeneri* and *S. Sonnei*. In Ozmert et al. study, there was no difference in clinical manifestations, except for seizures (16). The highest antibiotic resistance in the present study was related to SXT (89%) and ampicillin (77%). Several reports of high prevalence of resistance to ampicillin and SXT have been published (17, 18). According to our study and above-mentioned studies, SXT and ampicillin are not appropriate drugs to treat shigellosis. Quinolones and fluoroquinolones are most important groups of antibiotics, used in the treatment of shigellosis in various parts of the world. Unlike Xing et al. study in China, resistance to nalidixic acid in our study is much lower (12%) (17). This indicates that the optimum use of antibiotics as a treatment choice can prevent specific strains resistance. GU et al. showed that a considerable increase in resistance to ciprofloxacin was observed over the 12-year period in Asia–Africa countries whereas, in the United States and Europe resistance to third-generation cephalosporins was less than 1%. Resistance to third-generation cephalosporin, has been particularly found in Vietnam, China and Iran (3). Although, the studies have reported an increasing antibiotic resistance to ceftriaxone in *Shigella* species, but high resistance (51%) to ceftriaxone in our study is notable. Unlike our results, a study in Nepal from 2002 to 2004 showed that all *Shigella* isolated strains were susceptible to ceftriaxone (19). Also, a study from Peru reported the same results (10). Compared to previous studies in this center, resistance to ceftriaxone has dramatically increased (15). Ceftriaxone is currently the first-line treatment in shigellosis in hospitalized patients. Irregular use of antibiotics has led to the development of resistant strains. In this study, Multidrug Resistance (MDR) observed are as follows: ampicillin and co-trimoxazole (57.5%), ampicillin, ceftriaxone, co-trimoxazole and azithromycin (41.9%) and ampicillin, ciprofloxacin, co-trimoxazole and azithromycin (2.5%). In a systematic study, Ozmert et al. showed, that MDR was observed in 24% of cases of shigellosis, most of which were resistance to ampicillin and co-trimoxazole (16). Zhang et al. showed that approximately 80% of the isolates were resistant to at least eight anti-microbial agents, 14% to at least ten antimicrobials tested and 10 isolates to fourteen anti-microbials, including sulfonamides, fluoroquinolones, tetracycline’s, aminoglyco-sides and β-lactamases (17). The results highlighted the urgency of the treatment of shigellosis in health centers. MDR pathogens have made the treatment difficult. Ceftriaxone, ciprofloxacin, azithromycin and some antibiotics had been proposed for MDR shigella strains, by Erdogan et al. (20). The increasing prevalence of MDR to *Shigella* species is a serious threat, especially in developing countries with health and nutritional problems. Several reasons could be accounted for this situation: First, the quality of public health has a significant impact. Inappropriate health standards, low awareness of personal hygiene and poor quality water have an important role in transmission of *Shigella*. Second, some countries lack the infrastructure to monitor the use of antibiotics at the national level. Finally, guidelines for antibiotic treatment are limited, and arbitrary use of antibiotics has led to the creation of resistant strains of bacteria.

As a consequence of high resistance of *Shigella* species to ceftriaxone in this study, we suggest to not to be used as the first-line therapy. In addition, more extensive studies are required to identify other antibiotic resistant strains of *Shigella*.
